# Fondaparınux Versus Nadroparın for Preventıon of Venous Thromboembolısm After Electıve Hıp and Knee Arthroplasty^☆^

**DOI:** 10.1016/j.curtheres.2013.02.003

**Published:** 2013-06

**Authors:** Mahmut Argun, Mithat Oner, Mehmet Saglamoglu, Ibrahim Karaman, Ahmet Guney, Mehmet Halici, Ibrahim Halil Kafadar

**Affiliations:** Department of Orthopaedics and Traumatology, Erciyes University Medical Faculty, Kayseri, Turkey

**Keywords:** arthroplasty, fondaparinux, nadroparin, venous thromboembolism

## Abstract

**Objective:**

To evaluate the efficacy and safety of fondaparinux compared with nadroparin for prevention of venous thromboembolism after arthroplasty.

**Patients and methods:**

One hundred fifteen patients were randomized into 2 treatment groups. Patients were given fondaparinux in Group I and nadroparin in Group II. Measurements were performed on Days 1, 5, and 21. The wound area was assessed with a subjective visual analog scale.

**Results:**

The blood counts, clinical biochemical tests, and coagulation tests (ie, thrombin time, partial thromboplastin time, activated partial thromboplastin time, fibrinogen, prothrombin time–International Normalized Ratio, and antithrombin III activity) did not show statistically significant differences between Group I and Group II. In both study groups, anti-factor Xa activities increased significantly on the fifth and 21st day. The scores of the subjective visual analog scale showed significance on Day 21.

**Conclusions:**

Our results confirm the safety and efficacy of both fondaparinux and nadroparin for prophylaxis after major orthopedic surgery.

## Introduction

Venous thromboembolism (VTE), which presents clinically as deep vein thrombosis (DVT) and pulmonary embolism, is the most frequent serious complication after major orthopedic surgery of the lower extremities.^1,2^ At present, anticoagulant prophylactic treatments such as low-dose heparin, low-molecular-weight heparins (LMWHs), warfarin, or recombinant hirudin are recommended, but the frequency of venographically proven DVT still ranges from 16% to 30%.^3^

The development of LMWHs has added a new dimension to the clinical management of thrombotic disorders. LMWHs are prepared by the enzymatic or chemical depolymerization of standard heparin. The growing use of LMWH in the prophylaxis and treatment of acute thrombotic disorders emphasizes the need for characterization of the platelet effects of these anticoagulants.^4^

Fondaparinux (Arixtra; Organon Sanofi-Synthelabo LLC, Oss, the Netherlands) a pentasaccharide, is a selective inhibitor of coagulation factor Xa and interrupts the coagulation cascade by inhibiting thrombin generation without inactivating thrombin. This drug has been shown to be superior to LMWHs in the prevention of DVT after hip or knee replacement, and at least as effective and as safe as LMWHs and unfractionated heparin in patients with pulmonary embolism and DVT.^5,6^

Nadroparin calcium (Fraxipoarine; Sanofi, Winthrop, France) is an LMWH with a mean molecular weight of 4500 Daltons. Nadroparine calcium is a fractionated heparin derivative with a wide clinical spectrum of use as an anticoagulant for thromboprophylaxis to reduce the incidence of DVT.^7^

In our prospective, randomized study we aimed to assess the efficacy and safety of fondaparinux compared with nadroparin for prevention of VTE in patients undergoing elective hip and knee replacement surgery.

## Patients and Methods

### Patients

Patients of both sexes were eligible if they were aged 25 years or older and were scheduled for primary elective total hip and knee replacement surgery.

We excluded patients if bilateral surgery was planned during the same procedure or within 2 weeks after inclusion. We also excluded patients who had at least 1 of the following criteria: major orthopedic surgery within the previous 1 month; active bleeding; acute bacterial endocarditis; documented congenital or acquired bleeding disorder; current ulceration or angiodysplastic gastrointestinal disease; history of haemorrhagic stroke or myocardial infarction within the previous 6 months; brain, spinal, or ophthalmologic surgery within the previous 3 months; history of malignancy, DVT, or pulmonary embolism; planned indwelling intrathecal or epidural catheter for more than 6 hours after the end of surgery; hypersensitivity to heparin, LMWHs, porcine products, or iodinated contrast media; uncontrolled hypertension (ie, diastolic blood pressure >120 mm Hg, systolic blood pressure >200 mm Hg); platelet count <100×10^3^/μL; hepatic and renal insufficiency; or anticoagulant treatment or any type of anticoagulant or fibrinolytic therapy or dextran within the 2 days preceding the first administration of the study drug.

### Study design

This open, prospective, comparative randomized study was carried out in a university hospital medical center over a period of 1 year. A total of 115 patients were randomized into 2 treatment groups.

In Group I, patients were assigned to once-daily subcutaneous injections of 2.5 mg fondaparinux 6 hours before surgery. The first injection of fondaparinux took place a mean of 12 hours after the operation and subsequent injections of 2.5 mg fondaparinux was given once daily.

In Group II, the first subcutaneous injection of nadroparin calcium was given 12 hours before surgery, the second 12 hours after completion of surgery, and subsequent injections once daily in the morning. The dose of the nadroparin was arranged in accordance with the manufacturer’s recommendation.

The day of surgery was defined as Day 1 and the efficacy outcome was assessed on Day 5 and Day 21. During follow-up, patients were instructed to report any symptoms or signs of venous thromboembolism or bleeding, and any other clinical event that arose after treatment had been completed. If VTE arose during the study, treatment was left to the discretion of the investigator.

Intra- and postoperative bleeding was assessed, first according to the clinical impression of the surgeon (recorded as normal, greater than normal, or less than normal), and then by recording the blood loss collected by suction and swabs, drainage volume, and the number and amount of blood transfusions.

Throughout the treatment period, intermittent pneumatic compression, dextran, thrombolytic treatment, and any other anticoagulant agents were prohibited. The patients were advised to avoid taking aspirin or nonsteroidal anti-inflammatory drugs whenever possible. Use of graduated compression stockings and physiotherapy was recommended.

Measurements, including blood counts, clinical biochemical tests, and coagulation tests were done before the surgical procedure and on the fifth and 21st days after surgery. Blood counts included hemoglobin and heamatocrit levels and platelet counts; clinical biochemical tests included alanine aminotransferase, aspartate aminotransferase, blood urea nitrogen, creatinine, and direct and indirect bilirubin. Coagulation tests included thrombin time, partial thromboplastin time, activated partial thromboplastin time, fibrinogen, prothrombin time-International Normalized Ratio, antithrombin III activity, anti-factor Xa activity, and anti-IIa activity.

Bruising of the wound area was assessed by the same observer with use of a subjective visual analog scale described by Warwick et al^8^ on the fifth and 21st postoperative days. With regard to bruising, a score of 0 points indicated no discoloration, 1 point indicated light-yellow discoloration, 2 points indicated dark-yellow discoloration, 3 points indicated yellow discoloration greater than the area of 3 palms, 4 points indicated yellow-and-black discoloration, and 5 points indicated yellow-and-black discoloration greater than the area of 3 palms.

The study was done in accordance with the ethical principles in the Declaration of Helsinki. The ethics committee of the university approved the protocol, and written informed consent was obtained from all patients before randomization.

## Statıstical Analysıs

All statistical analysis was performed using SPSS 10.0 for Windows (IBM Corp, Armonk, New York). A *P* value of 0.05 or less was considered statistically significant. The outcomes were compared with use of the *t* test, the χ^2^ test, the Mann-Whitney U test with a Bonferroni correction, and repeated measures analysis of variance test as appropriate.

## Results

A total of 115 patients were randomized to receive either fondaparinux or nadroparin into 2 treatment groups over a period of 1 year.

Thirty-eight of 60 patients in Group I underwent total hip arthroplasty (THA), remaining 22 underwent total knee arthoplasty (TKA). Two THA and 3 TKA patients were excluded from the study due to the postoperative hematoma that was treated with drainage. Twenty-eight of 55 patients in Group II underwent THA, the remaining 27 underwent TKA. Two of the TKA patients were excluded from the study due to postoperative wound hematoma that was treated with drainage. There was not a significant difference between the groups in terms of hematoma frequency (*P* > 0.05). Serious adverse effects like thrombocytopenia, anemia, and allergy were not seen in either group. The remaining 55 (91.7%) patients in Group I and 53 (96.4%) patients in Group II completed the study (*P* > 0.05).

Baseline characteristics, including age, sex distribution of the patients, and the number of patients who had systemic disease like diabetes and hypertension were similar in both groups (see Table I) (*P* > 0.05).

The intraoperative blood loss, whether judged clinically or according to aspirated volume, and the mean volume of intraoperative blood transfusion did not show statistically significant differences in either group (*P >* 0.05).

When we evaluated the blood counts, including hemoglobin and hematocrit levels and platelet counts, we found that there was no statistically significant difference between groups.

The results of clinical biochemical tests and coagulation tests are presented in Table II and the results of coagulation tests are shown in Table III.

Clinical biochemical tests, including alanine aminotransferase, aspartate aminotransferase, blood urea nitrogen, creatinine, direct and indirect bilirubin, and coagulation tests, including thrombin time, partial thromboplastin time, activated partial thromboplastin time, fibrinogen, prothrombin time, prothrombin time-International Normalized Ratio, and antithrombin III activity did not show statistically significant differences between Group I and Group II (*P* > 0.05). In both study groups, antifactor Xa activities increased significantly on the fifth and 21st day (*P <* 0.05). The antifactor Xa activity was higher in the fondaparinux group than the nadroparin group. Although the anti-IIa activity did not show significance between days (*P >* 0.05), it showed significance between groups on Day 1, Day 5, and Day 21 (*P* < 0.05). Anti-IIa activity was significantly higher in nadroparin group (*P* < 0.05).

When we evaluated the scores of the subjective visual analog scale, although there was not a statistically significant difference between groups on the fifth day, we found significance on Day 21 (Table IV).

Only 1 proximal DVT occurred in a patient in Group II at the 19th day, which was confirmed by Doppler ultrasonography. None of the patients in the study developed major bleeding during the study period.

## Discussion

VTE is a common, serious complication after major orthopedic surgery of the lower extremities.^1,2^ Because of the high risk of VTE and its considerable morbidity and mortality after major orthopedic surgery of the lower extremities, the routine use of VTE prophylaxis is recommended for all patients undergoing hip and knee arthroplasty.^2^

Studies of the time course of VTE events after THA and TKA have also demonstrated that when VTE prophylaxis is used, the period over which patients are at risk is extended beyond 1 week, and events are likely to occur after a patient is discharged from the hospital.^2,9^ A retrospective study in a large group found that 76% of patients who had undergone THA and 47% who had undergone TKA experienced a VTE event after being discharged from the hospital.^2^ Median time of diagnosis of thromboembolism was 17 days with THA and 7 days with TKA. In our study, we followed patients for 3 weeks, which included the period over which patients are at high risk.

A meta-analysis of fondaparinux Phase III data in 5385 patients showed that, in patients who have had major orthopedic surgery, fondaparinux reduced the incidence of VTE to 6.8% and this result was better than the results of enoxaparin (13.7%).^10^

Nadroparin thromboprophylaxis is known to reduce the incidence of DVT by 87%. This major decrease in DVT incidence was not associated with an increased risk of bleeding.^11^

In the Evaluation of AriXtra for the Prevention of vEnous thRomboembolism in daily pracTice study^12^ it was reported that the overall rate of major bleeding with fondaparinux prophylaxis was 0.8%, of which 71.4% of cases occurred within 15 days after surgery. Fatal bleeding and bleeding in a critical organ was observed in 0.1% of patients. Bleeding at the surgical site leading to reoperation occurred in 0.5% of patients and bleeding at a nonsurgical site necessitating the transfusion of >2 units of blood occurred in 0.1% of patients. In our small study group, bleeding at the surgical site leading to drainage due to postoperative hematoma occured in 8.3% of patients in the fondaparinux group and 3.6% in the nadroparin group. Although the percentage was high in the fondaparinux group there was not a statistically significant difference between the 2 groups. None of the patients in the 2 groups had bleeding that was fatal or involved a critical organ.

In a Phase III, long-term prophylactic study in hip fracture surgery, it was reported that there were no differences in the overall incidence of adverse events and in platelet count between the fondaparinux and placebo groups. However, until the safety of fondaparinux concerning immune thrombocytopenia is firmly established by clinical experience, periodic blood platelet count monitoring is recommended during the course of fondaparinux therapy.^13^ Similarly, an increase in serum transaminases was rare in both groups.^14^

In our study, there were no differences between fondaparinux and nadroparin groups in the blood counts, including hemoglobin and platelet counts. When we evaluated the serum transaminases, although there was a slight increase in the mean value of AST on the fifth and 21st day in both groups, the difference showed no significance.

It is known that LMWH does not usually elevate activated partial thromboplastin time; it is valued for its antithrombotic rather than its anticoagulant effect.^15^ None of the coagulation tests showed significant changes in either group of our study. In general, antifactor Xa assays are more sensitive to the effects of LMWHs and are therefore more useful in this respect than anti-IIa assays.^16,17^ Differences were also observed in the antifactor Xa activities of the LMWHs. All the LMWHs studied had weaker antifactor Xa potency than standard heparin. We found that antifactor Xa activities were increased significantly on the fifth and 21st day in our both study groups.

Regarding the subjective visual analog scale, there was significantly less bruising and oozing on the 21st day in the nadroparin group than in the fondapainux group. The clinical importance of these differences is unclear because it is not known how they are related to the cost of the dressings, the duration of hospitalization, the formation of hematomas, or the rate of infection. Furthermore, the scales for the measurement of bruising and oozing were subjective and the examination depends on the observer. Unless these side effects were not to be associated with the disastrous complication of infection, it would not be viewed as a disadvantage.

In our prospective study, symptomatic VTE events were rare, during both the treatment and follow-up periods. Our results confirm the safety and efficacy of both fondaparinux and nadroparin for prophylaxis after major orthopedic surgery of the lower extremities and showed that neither drug is associated with a risk of major bleeding complications.

## Figures and Tables

**Table I:** Distribution of patients by age, sex, and systemic disease.*

	Group I	Group II	*P*
Age, y	58.7 (13.6)	60.0 (8.4)	>0.05
Women	34 (61.8)	33 (62.2)	>0.05
Men	21 (38.2)	20 (37.8)	>0.05
Diabetes mellitus	5 (9.1)	4 (7.5)	>0.05
Hipertension	12 (21.8)	10 (18.9)	>0.05

⁎ Except for age, which is presented as mean (SD), values are given as n (%).

**Table II:** The results of clinical biochemical tests of both groups on Day 1, 5, and 21.*

Test	Day 1	Day 5	Day 21	F	*P*†	*P*‡	*P*§	*P*∥
BUN, mg/dL								
Group I	16.7 (6.6)	17.8 (11.4)	17.1 (8.0)	6.7	0.07	0.26	0.09	0.29
Group II	14.8 (3.9)	14.2 (3.7)	14.9 (4.4)	1.08	0.35			
Creatinin, mg/dL								
Group I	0.78 (0.2)	0.9 (0.57)	0.95 (0.5)	1.5	0.24	0.30	0.30	0.10
Group II	0.74 (0.1)	0.78 (0.1)	0.7 (0.16)	2.4	0.10			
AST, U/I								
Group I	24.8 (6.0)	31.5 (12.4)	26.3 (9.4)	2.8	0.08	0.07	0.06	0.15
Group II	23.0 (4.9)	26.2 (7.1)	21.6 (6.1)	1.2	0.3			
ALT, U/I								
Group I	21.5 (14.3)	18.6 (9.4)	20.8 (8.8)	0.54	0.60	0.7	0.06	0.11
Group II	22.6 (6.2)	23.7 (10.0)	25.2 (6.9)	2.02	0.15			
Tbil, mg/dL								
Group I	0.76 (0.25)	0.78 (0.17)	0.86 (0.27)	1.4	0.26	0.23	0.97	0.09
Group II	0.72 (0.26)	0.75 (0.3)	0.71 (0.2)	5.7	0.11			
Ibil, mg/Dl								
Group I	0.19 (0.96)	0.21 (0.11)	0.24 (0.16)	6.0	0.05	0.58	0.62	0.06
Group II	0.20 (0.91)	0.23 (0.14)	0.21 (0.1)	6.2	0.07			

AST, aspartate aminotransferase; BUN, blood urea nitrogen; Ibil, indirect bilirubin; Tbil, total bilirubin.

⁎ Values for Days 1, 5, and 21 are given as mean (SD).

† Value for the comparison of a parameter in a group between the days.

‡ Value for the comparison of a parameter between groups on Day 1.

§ Value for the comparison of a parameter between groups on Day 5.

∥ Value for the comparison of a parameter between groups on Day 21.

**Table III:** Results of coagulation tests of both groups on Day 1,5, and 21.*

Test	Day 1	Day 5	Day 21	F	*P*†	*P*‡	*P*§	*P*∥
TT, sec						0.06	0.90	0.88
Group I	12.5 (0.7)	12.3 (1.0)	12.6 (0.9)	2.2	0.13			
Group II	12.1 (0.4)	12.4 (0.6)	12.5 (0.7)	2.5	0.09			
PTZ, sec						0.40	0.45	0.70
Group I	11.9 (0.9)	12.2 (0.7)	12.4 (0.5)	5.1	0.16			
Group II	12.0 (0.8)	12.3 (1.2)	12.5 (0.7)	6.1	0.06			
aPTT, sec						0.27	0.36	0.11
Group I	28.7 (3.6)	29.5 (3.6)	29.4 (2.9)	0.6	0.56			
Group II	27.7 (1.7)	28.3 (2.9)	27.4 (4.7)	6.4	0.06			
PT-INR						0.56	0.18	0.14
Group I	0.94 (0.11)	0.95 (0.2)	0.96 (0.09)	4.0	0.23			
Group II	0.95 (0.08)	0.97 (0.1)	0.98 (0.1)	5.3	0.08			
Fibrinogen, mg/dL						0.60	0.08	0.95
Group I	407.0 (69.4)	606.0 (131.0)	592.0 (125.0)	36.0	0.07			
Group II	398 (94.6)	595.0 (152.0)	594.0 (197.0)	27.7	0.11			
AT-III, %						0.45	0.26	0.06
Group I	110.0 (10.4)	108.5 (12.6)	111.5 (12.5)	0.02	0.98			
Group II	112.7 (13.0)	113.7 (22.4)	109.0 (19.5)	7.5	0.09			
Anti-IIa, %						0.03	0.02	0.04
Group I	83.8 (26.2)	80.6 (17.5)	86.5 (23.2)	4.4	0.10			
Group II	98.2 (17.7)	103.6 (23.3)	101.8 (18.6)	4.3	0.13			
Anti-Xa, U/mL						0.13	0.35	0.99
Group I	0.03 (0.04)	0.2 (0.12)	0.19 (0.10)	34.1	0.01			
Group II	0.01 (0.006)	0.16 (0.12)	0.18 (0.13)	21.7	0.01			

Anti-FIIa, antifactor IIa; Anti-Xa, anticoagulation factor Xa; aPTT, activated partial thromboplastin time; AT III, antithrombin III; PT INR, prothrombin time-International Normalized Ratio; PTZ, partial thromboplastin time; TT, thrombin time.

⁎ Values for Days 1, 5, and 21 are given as mean (SD).

† Value for the comparison of a parameter in a group between the days.

‡ Value for the comparison of a parameter between groups on Day 1.

§ Value for the comparison of a parameter between groups on Day 5.

∥ Value for the comparison of a parameter between groups on Day 21.

**Table IV:** Visual analog scale scores.

Group	n	Day 5 (0-5)	Day 21 (0-5)	P*
Group I	55	2.63	2.61	0.58
Group II	53	2.76	1.43	0.01
*P*†		0.31	0.01	

⁎ Value for the comparison of the score in a group between Days 5 and 21.

† Value for the comparison of the score between groups.
